# Differential adipokine receptor expression on circulating leukocyte subsets in lean and obese children

**DOI:** 10.1371/journal.pone.0187068

**Published:** 2017-10-26

**Authors:** Genoveva Keustermans, Laila B. van der Heijden, Berlinda Boer, Rianne Scholman, Roos Nuboer, Gerard Pasterkamp, Berent Prakken, Wilco de Jager, Eric Kalkhoven, Arieke J. Janse, Henk S. Schipper

**Affiliations:** 1 Laboratory for Translational Immunology, University Medical Center Utrecht, Utrecht, The Netherlands; 2 Division of Pediatrics, Hospital Gelderse Vallei, Ede, The Netherlands; 3 Division of Pediatrics, Meander Medical Centre, Amersfoort, The Netherlands; 4 Department of Experimental Cardiology, University Medical Center Utrecht, Utrecht, The Netherlands; 5 Division of Pediatrics, Wilhelmina Children’s Hospital, University Medical Center Utrecht, Utrecht, The Netherlands; 6 Molecular Cancer Research and Center for Molecular Medicine, University Medical Center Utrecht, Utrecht, The Netherlands; 7 Department of Pediatric Cardiology, Wilhelmina Children’s Hospital, University Medical Center Utrecht, Utrecht, The Netherlands; University of Bergen, NORWAY

## Abstract

**Background:**

Childhood obesity prevalence has increased worldwide and is an important risk factor for type 2 diabetes (T2D) and cardiovascular disease (CVD). The production of inflammatory adipokines by obese adipose tissue contributes to the development of T2D and CVD. While levels of circulating adipokines such as adiponectin and leptin have been established in obese children and adults, the expression of adiponectin and leptin receptors on circulating immune cells can modulate adipokine signalling, but has not been studied so far. Here, we aim to establish the expression of adiponectin and leptin receptors on circulating immune cells in obese children pre and post-lifestyle intervention compared to normal weight control children.

**Methods:**

13 obese children before and after a 1-year lifestyle intervention were compared with an age and sex-matched normal weight control group of 15 children. Next to routine clinical and biochemical parameters, circulating adipokines were measured, and flow cytometric analysis of adiponectin receptor 1 and 2 (AdipoR1, AdipoR2) and leptin receptor expression on peripheral blood mononuclear cell subsets was performed.

**Results:**

Obese children exhibited typical clinical and biochemical characteristics compared to controls, including a higher BMI-SD, blood pressure and circulating leptin levels, combined with a lower insulin sensitivity index (QUICKI). The 1-year lifestyle intervention resulted in stabilization of their BMI-SD. Overall, circulating leukocyte subsets showed distinct adipokine receptor expression profiles. While monocytes expressed high levels of all adipokine receptors, NK and iNKT cells predominantly expressed AdipoR2, and B-lymphocytes and CD4^+^ and CD8^+^ T-lymphocyte subsets expressed AdipoR2 as well as leptin receptor. Strikingly though, leukocyte subset numbers and adipokine receptor expression profiles were largely similar in obese children and controls. Obese children showed higher naïve B-cell numbers, and pre-intervention also higher numbers of immature transition B-cells and intermediate CD14^++^CD16^+^ monocytes combined with lower total monocyte numbers, compared to controls. Furthermore, adiponectin receptor 1 expression on nonclassical CD14^+^CD16^++^ monocytes was consistently upregulated in obese children pre-intervention, compared to controls. However, none of the differences in leukocyte subset numbers and adipokine receptor expression profiles between obese children and controls remained significant after multiple testing correction.

**Conclusions:**

First, the distinct adipokine receptor profiles of circulating leukocyte subsets may partly explain the differential impact of adipokines on leukocyte subsets. Second, the similarities in adipokine receptor expression profiles between obese children and normal weight controls suggest that adipokine signaling in childhood obesity is primarily modulated by circulating adipokine levels, instead of adipokine receptor expression.

## Introduction

Obese children often remain obese in adulthood, and are at risk for metabolic syndrome and cardiovascular disease later in life [1–3]. Over the last few decades, enhanced excretion of inflammatory adipose tissue derived proteins (adipokines) emerged as one of the mechanisms underlying the cardiometabolic sequelae in obesity [4,5]. The adipokine profile in childhood and adulthood obesity includes increased levels of the inflammatory adipokines chemerin and leptin, and decreased levels of the anti-inflammatory adiponectin, which together propagates systemic inflammation, insulin resistance and vascular dysfunction, as a precursor for cardiovascular disease [4,6–11].

The impact of circulating adipokines is not merely determined by plasma levels, but also orchestrated by differential adipokine receptor expression on target organs. For example, adiponectin receptor 1 (AdipoR1) and receptor 2 (AdipoR2) are significantly homologous (67% amino acid identity), and both serve as a receptor for globular and full-length adiponectin [12]. However, AdipoR1 is predominantly expressed in liver, skeletal muscle, macrophages and hypothalamus, while AdipoR2 is most abundant in liver, white adipose tissue, and the vasculature. The differential tissue distribution and downstream signalling pathways of AdipoR1 and AdipoR2 importantly contribute to the plethora of adiponectin’s biological actions [10]. Next to differences in tissue distribution, up/downregulation of adipokine receptor expression under specific conditions can also modulate adipokine effects. Natural Killer (NK) cells, for instance, critically depend on leptin receptor expression for their activation and function [13]. While leptin receptor-deficient mice showed impaired NK cell activity, leptin receptor expression was upregulated in rats with diet-induced obesity, apparently to compensate for decreased downstream signalling [14]. Taken together, both tissue distribution and disease-specific up/downregulation of adipokine receptors can modulate adipokine effects.

Whereas studying adipose tissue, liver and vascular distribution of adipokine receptors is precluded for medical ethical reasons, circulating leukocytes are readily available and play a pivotal role in systemic inflammation in obesity [15–18]. Here, we used recently available flow cytometry antibodies to study the expression of AdipoR1, AdipoR2 and leptin receptor on circulating leukocyte subsets, in addition to measuring circulating adipokine levels. Considering the pivotal role of adipokine signalling in obesity, we included obese children pre and post-lifestyle intervention, next to normal weight control children. This study thus aims to unravel the differential impact of circulating adipokine levels and adipokine receptor expression on adipokine signalling in childhood obesity.

## Materials and methods

### Patients

This observational cohort study included 15 lean control children and 13 obese children aged 4–18 years, all patients of the pediatric outpatient department of Meander Medical Centre in Amersfoort and Hospital Gelderse Vallei Hospital in Ede, The Netherlands. Body Mass Index standard deviation (BMI-SD) values were calculated based on results of the Fifth Dutch Growth Study [19]. Obesity was defined using established international age and sex-specific BMI cut-off points [20]. The obese patients were enrolled in an established 1-year multidisciplinary, multicomponent, family-based treatment programme developed in the Gelderse Vallei Hospital in Ede, The Netherlands [21]. Patients were included from 2010 to 2015. Anthropometric measurements, blood pressure (BP) measurements and laboratory samples were collected at baseline (lean controls, obese children), and at the end of the 1-year intervention program (obese children). The study was approved by the ethical committee of Wageningen UR (METC 12/26) and the University Medical Center Utrecht (METC 09/217K). Written informed consent was obtained from children older than 12 years and from the parents.

### Clinical variables

Waist circumference was used as a marker of central adiposity and measured with a flexible tape to the nearest 0.1 cm at umbilicus height. Blood pressure (BP) was measured in supine position with an automated blood pressure monitor (Welch Allyn VSM 300, Skaneateles Falles, NY, USA) after 5 minutes of rest during a well visit in the outpatient clinic. A minimum of two BP measurements was performed, with an interval of at least one minute between the measurements. The mean of these two measurements was collected for data analysis. Blood pressure percentile scores were obtained according to the Fourth Report on BP in children [22].

### Routine laboratory measurements

Routine laboratory testing included fasting glucose, insulin levels and lipid profiles (total cholesterol, high-density lipoprotein [HDL] cholesterol, low-density lipoprotein [LDL], and triglycerides), as well as samples for leukocyte differentiation, and alanine-aminotranspherase (ALT). The insulin sensitivity index (QUICKI) was calculated according to international standards [23].

### Flow cytometry

Whole blood samples collected in sodium heparin tubes (BD vacutainer 367876) where spun down at room temperature, 160g for 10 minutes. Plasma was subsequently removed and stored at -80°C awaiting further use. Peripheral Blood mononuclear cells (PBMC) were isolated using Ficoll-Paque density gradient centrifugation and samples were stored in fetal bovine serum (FBS) (Biowest) supplemented with 10% DMSO (Sigma-Aldrich) at -150°C. All children donated 6–12ml whole blood, with 6–10 million PBMC per 6ml whole blood sample. Upon preparation for flow cytometry, the stored PBMC samples where thawed and washed in medium comprising of RPMI1640 supplemented with l-glutamate and 25 mM HEPES (Gibco), containing 2% FBS (Biowest) and penicillin/streptomycin (100 U/mL) (Invitrogen). Cells were spun down for 10 minutes at 280g at room temperature. All samples were stained for 20 minutes in the dark at 4°C and subsequently analyzed on the BD LSR Fortessa. For monocyte, NK cell, B-cell, T-helper, T-effector/memory and Treg phenotyping, 200.000 PBMCs were analyzed per sample. For iNKT cell phenotyping, 750.000 PBMCs were analyzed per sample. The following antibodies were used: CD3 AF700 (Biolegend, clone UCHT1), CD4 PerCP-Cy5.5 (BD, clone SK3), CD25 PE-Cy7 (BD, clone M-A251), CD45RO BV711 (Biolegend, clone UCHL1), CD127 BV421 (BD Horizon, clone HIL-7R-M21), CD8 V500 (BD, clone RPA-T8), Leptin receptor Alexaflour647 (BD,clone 52263), ADIPOR1 FITC (USBio, rabbit polyclonal antibody). ADIPOR2 PE (USBio, rabbit polyclonal antibody), CD27 APC-eFluor780 (eBioscience, clone O323), CD28 BV421 (BD Horizon, clone CD28.2), CCR6 PE-Cy7 (eBiosciences, clone R6H1), CXCR3 BV510 (Biolegend, clone GO25H7), CD16 V500 (BD Horizon, clone 3G8), CD56 PE-Cy7 (BD, clone NCAM16.2), CD1d tetramer BV421 (NIH, hCD1d-PBS-57), CD10 PE-Cy7 (BD, clone HI10A), CD19 APC-efluor 780 (eBioscience, clone HIB19), CD21 BV711 (BD, clone B-ly4), CD27 BV510 (BD Horizon, clone L128), CD38 PerCP-Cy5.5 (BD Pharmigen, clone HIT2). Leukocyte subset numbers were calculated using the differential blood count (e.g. number of CD14^++^CD16^+^ monocytes = (fraction of CD14^++^CD16^+^ monocytes / total monocytes) x differential blood count monocyte number). Gating strategy of the leukocyte subsets is shown in the S1 Fig.

### Multiplex immune assay (MIA)

Plasma levels of adiponectin, chemerin and leptin were measured by a MIA using Luminex xMAP technology (xMAP, Luminex Austin TX USA) validated by the Laboratory of Translational Immunology, University Medical Center Utrecht [24]. Biorad FlexMAP3D (Biorad laboratories. Hercules USA) and xPONENT software version 4.2 (Luminex) were used for acquisition and data was analyzed by 5-parametric curve fitting using Bio-Plex Manager software, version 6.1.1 (Biorad).

### Statistical analysis

Differences between groups were studied with an independent-sample Student’s t-test for normally distributed data, and with a Mann-Whitney U test for non-parametric comparisons. Multiple testing correction using the Benjamini and Hochberg False Discovery Rate (FDR) procedure was applied when assessing leukocyte subset numbers and comparing adipokine receptor expression of leukocyte subsets in lean versus obese children pre and post-intervention. Pearson’s correlation coefficients were calculated to determine the correlation between levels of circulating adipokines and the expression of adiponectin and leptin receptors on circulating immune cells. Statistical analyses were performed with the SPSS 22 statistical package (IBM SPSS Statistics Inc, Chicago, IL, USA).

## Results

### Circulating leukocyte subset numbers

Obese children exhibited typical clinical and biochemical characteristics compared to age and sex-matched normal weight controls, with a higher BMI-SD, waist circumference and blood pressure, combined with a lower insulin sensitivity index (QUICKI) and higher plasma levels of alanine aminotransferase, chemerin and leptin (Table 1). The obese children participated in an established lifestyle intervention program [21], which resulted in stabilization of their BMI-SD and other clinical and biochemical characteristics, and enabled sampling pre and post-lifestyle intervention (Table 1).

**Table 1:** Patient characteristics.

	Lean controls (n = 15)	Obese—pre (n = 13)	Obese—post (n = 13
**Girls (number, %)**	8 (53.3)	7 (53.8)	7 (53.8)
**Age (years)**	11.7 ± 2.9	10.7 ± 3.9	12.0 ± 3.9
**BMI**	18.5 ± 2.6 *#	27.9 ± 5.2 *	28.2 ± 5.5 #
**BMI-SD**	0.5 ± 0.9 *#	3.3 ± 0.7 *	3.1 ± 0.7 #
**Waist (cm)**	64.5 ± 7.5 *#	94.4 ± 16.8 *	98.4 ± 17.0 #
**Systolic blood pressure (SBP)**	102.2 ± 12.7 *#	113.2 ± 12.2 *	114.8 ± 11.7 #
**SBP percentile**	39.9 ± 23.6 *#	65.7 ± 25.1 *	65.5 ± 28.4 #
**Diastolic blood pressure (DBP)**	57.7 ± 8.2 *#	67.2 ± 9.5 *	69.4 ± 10.5 #
**DBP percentile**	35.9 ± 19.9 *#	62.6 ± 23.9 *	65.4 ± 27.1 #
**QUICKI**	0.4 ± 0.0 *#	0.3 ± 0.0 *	0.3 ± 0.0 #
**Alanine aminotransferase (U/l)**	18.0 (14.5–21.8) *#	29.5 (22.2–53.9) *	30.3 (22.9–41.2) #
**Triacylglycerol (mmol/l)**	0.6 ± 0.3 *	1.0 ± 0.4 *	1.0 ± 0.7
**Total cholesterol (mmol/l)**	4.0 ± 0.8	4.0 ± 0.8	3.9 ± 0.9
**HDL-cholesterol (mmol/l)**	1.7 ± 0.9	1.2 ± 0.3	1.3 ± 0.2
**LDL-cholesterol (mmol/l)**	2.2 ± 0.7	2.3 ± 0.7	2.2 ± 0.8
**Adiponectin (ug/ml)**	18.9 (15.6–26.5)	12.9 (9.5–21.3)	13.6 (10.4–19.8)
**Chemerin (ug/ml)**	1.2 (1.0–1.7) *#	1.9 (1.8–2.7) *	2.3 (1.8–2.5) #
**Leptin (ng/ml)**	125 (16–195) *#	399 (219–692) *	411 (308–600) #

Clinical characteristics and laboratory parameters for lean controls versus obese children pre-lifestyle intervention (pre) and post-lifestyle intervention (post). Normally distributed data are shown as mean ± SD, non-parametric data as median (interquartile range).

* p<0.05 for lean controls compared to obese-pre.

# p<0.05 for lean controls versus obese-post.

**Fig 1:**
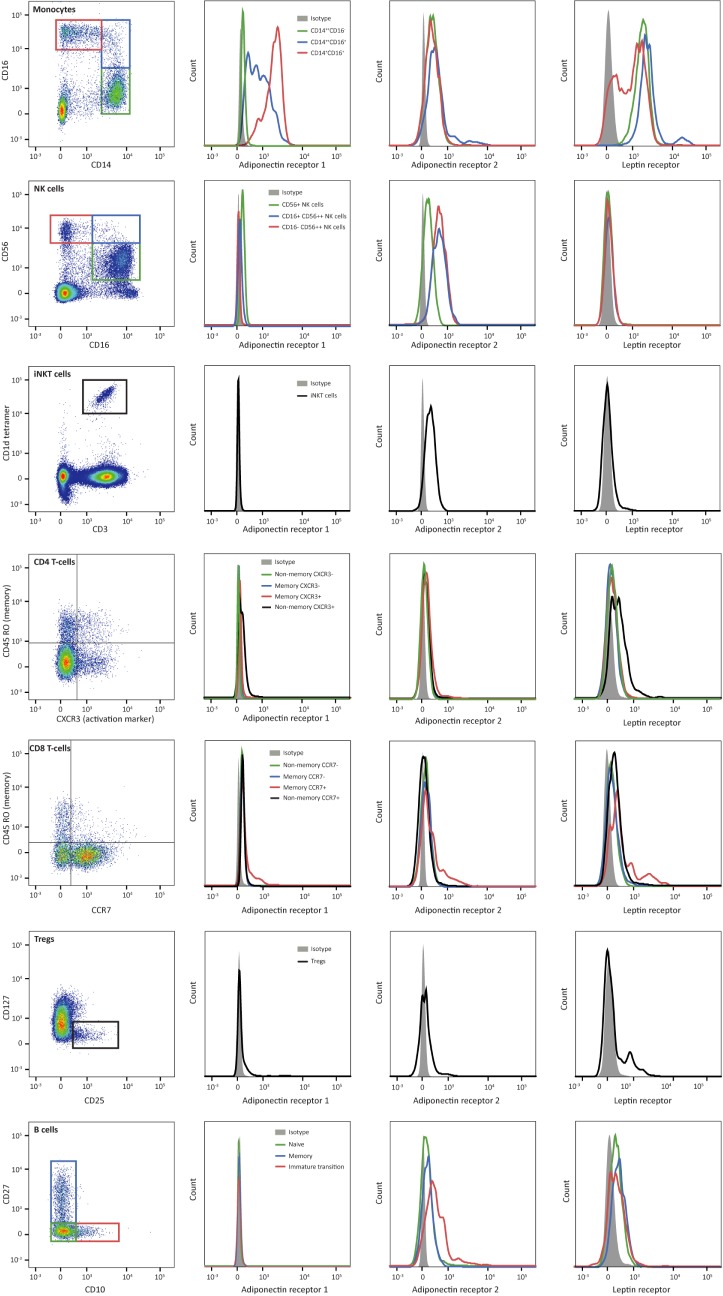
Adipokine receptor expression on leukocyte subsets. The first column illustrates the gating strategy for the leukocyte subsets. The other columns show representative histograms of the AdipoR1, AdipoR2 and leptin receptor expression of the different leukocyte subsets.

Circulating leukocytes were analysed with multi-parameter flow cytometry, and leukocyte subsets were gated according to international standards (Fig 1, S1 Fig) [25]. Overall, leukocyte subset numbers were comparable between obese children and lean controls. However, obese children pre and post-intervention showed higher naïve B-cell numbers, and obese children pre-intervention showed higher numbers of immature transition B-cells and intermediate CD14^++^CD16^+^ monocytes than lean controls (Table 2). Obese children pre-intervention additionally showed lower total monocyte numbers than lean controls. Notably, differences in leukocyte subset numbers did not survive multiple testing correction.

**Table 2:** Leukocyte subset numbers.

	Lean controls	Obese-pre	Obese-post
***Innate immunity***			
**Monocytes (total)**	400 (300–600) *	300 (300–400) *	400 (300–500)
**CD14^++^CD16^-^**	352 (255–542)	244 (196–303)	330 (245–374)
**CD14^++^CD16^+^**	15 (11–19)*	16 (13–22)*	18 (13–39)
**CD14^+^CD16^++^**	37 (33–94)	39 (30–79)	40 (36–69)
**Natural Killer cells (CD16^+^CD56^+^)**	150 (80–211)	224 (116–321)	226 (134–287)
**CD16^+^CD56^++^**	8.7 (5.5–12)	8.0 (4.9–13)	9.3 (5.8–12)
**CD16^-^CD56^++^**	4.9 (2.8–8.0)	5.5 (3.4–8.1)	5.0 (4.4–8.9)
***Bridging immunity***			
**Natural Killer T cells**	1.2 (0.6–2.2)	1.4 (0.9–3.9)	1.2 (1.0–3.6)
***Adaptive immunity***			
**B cells**			
**Naive (CD10^-^CD27^-^)**	178 (132–270) *#	255 (189–454) *	316 (224–454) #
**Memory (CD10^-^CD27^+^)**	48 (41–73)	69 (45–113)	74 (56–117)
**Immature transition (CD10^+^CD27^+^)**	10 (5.4–17) *	23 (9.3–39) *	15 (10–31)
**CD4^+^ T helper cells**			
**CD45RO^-^ CXCR3^-^**	511 (451–700)	639 (401–750)	624 (334–937)
**CD45RO^-^ CXCR3^+^**	58 (40–176)	58 (32–110) $	85 (68–162) $
**CD45RO^+^ CXCR3^-^**	140 (115–196)	151 (125–227)	207 (103–251)
**CD45RO^+^ CXCR3^+^**	111 (84–134)	67 (43–115)	84 (57–117)
**CD8+ cytotoxic T cells**			
**CD45RO^-^ CCR7^-^**	76 (37–138)	76 (43–178)	94 (29–161)
**CD45RO^-^ CCR7^+^**	284 (275–358)	355 (272–434)	294 (238–477)
**CD45RO^+^ CCR7^-^**	82 (44–85)	56 (38–91)	52 (42–73)
**CD45RO^+^ CCR7^+^**	15 (12–19)	13 (8.6–30)	18 (13–22)
**Regulatory T cells (CD25^+^CD127^-^)**	26 (14–35)	21 (16–28)	22 (13–33)

Leukocyte subset numbers (x 10^6^) of lean controls compared to obese children pre-lifestyle intervention (pre) and post-lifestyle intervention (post). Data are presented as median (interquartile range).

* p<0.05 for lean controls compared to obese-pre.

# p<0.05 for lean controls versus obese-post.

$ p<0.05 for obese-pre compared to obese-post.

### Adipokine receptor expression on leukocyte subsets

Focussing on the innate immune cells, monocytes expressed high levels of adipokine receptors compared to other circulating leukocyte subsets (Fig 1). Whereas nonclassical CD14^+^CD16^++^ monocytes particularly expressed high levels of AdipoR1, all monocyte subsets expressed AdipoR2, and classical CD14^++^CD16^-^ and intermediate CD14^++^CD16^+^ monocytes particularly expressed high leptin receptor levels. In contrast, Natural Killer (NK) cells and invariant Natural Killer T-cells (iNKT) predominantly showed AdipoR2 expression, next to discrete AdipoR1 expression by CD16^+^ NK cells (Fig 1, Tables 3, 4 and 5).

**Table 3:** Adiponectin receptor 1 expression.

	Lean controls	Obese-pre	Obese-post
***Innate immunity***			
**Monocytes (total)**	21 (18–31)	31 (18–36)	25 (20–29)
**CD14^++^CD16^-^**	10 (8.8–16) #	8.2 (4.6–27)	7.7 (3.7–11) #
**CD14^++^CD16^+^**	82 (68–88)	90 (81–94)	84 (73–93)
**CD14^+^CD16^++^**	84 (71–92) *	94 (77–98) *	91 (85–96)
**Natural Killer cells (CD16^+^CD56^+^)**	82 (56–92)	86 (76–95)	88 (78–95)
**CD16^+^CD56^++^**	41 (29–63)	51 (39–58)	56 (44–64)
**CD16^-^CD56^++^**	1.6 (0.5–3.1)	0.9 (0.1–4.6)	0.8 (0.4–1.8)
***Bridging immunity***			
**Natural Killer T cells**	7.6 (4.1–25)	12 (4.1–18)	6.9 (5.0–15)
***Adaptive immunity***			
**B cells**			
**Naive (CD10^-^CD27^-^)**	1.1 (0.6–2.2) #	0.8 (0.5–2.0)	0.5 (0.4–1.0) #
**Memory (CD10^-^CD27^+^)**	5.9 (3.8–6.6)	5.6 (3.8–8.8)	6.4 (3.5–9.6)
**Immature transition (CD10^+^CD27^+^)**	0.0 (0.0–0.2) *	0.7(0.3–1.0) *$	0.1 (0.0–0.5) $
**CD4^+^ T helper cells**			
**CD45RO^-^ CXCR3^-^**	5.2 (1.6–21)	3.3 (1.0–22)	3.6 (1.6–19)
**CD45RO^-^ CXCR3^+^**	64 (45–92)	47 (37–71)	63 (49–81)
**CD45RO^+^ CXCR3^-^**	6.1 (2.1–12)	4.7 (1.6–12)	6.6 (2.2–11)
**CD45RO^+^ CXCR3^+^**	24 (12–38)	21 (12–42)	41 (16–56)
**CD8+ cytotoxic T cells**			
**CD45RO^-^ CCR7^-^**	46 (41–55)	51 (42–61)	50 (42–61)
**CD45RO^-^ CCR7^+^**	60 (55–74)	54 (45–66)	53 (46–68)
**CD45RO^+^ CCR7^-^**	34(30–43)	36 (31–52)	38 (34–43)
**CD45RO^+^ CCR7^+^**	70 (66–77)	68 (52–90)	72 (67–87)
**Regulatory T cells (CD25^+^CD127^-^)**	3.9 (1.6–14)	4.0 (1.0–9.0)	4.8 (1.6–9.7)

Percentage expression of adiponectin receptor 1 on leukocyte subsets of lean controls compared to obese children pre-lifestyle intervention (pre) and post-lifestyle intervention (post). Data are presented as median (interquartile range).

* p<0.05 for lean controls compared to obese-pre.

# p<0.05 for lean controls versus obese-post.

$ p<0.05 for obese-pre compared to obese-post.

**Table 4:** Adiponectin receptor 2 expression.

	Lean controls	Obese-pre	Obese-post
***Innate immunity***			
**Monocytes (total)**	60 (32–67)	68 (46–80)	57 (43–70)
**CD14^++^CD16^-^**	59 (29–66)	60 (45–80)	54 (41–67)
**CD14^++^CD16^+^**	79 (60–88)	80 (67–93)	75 (59–86)
**CD14^+^CD16^++^**	60 (44–70)	63 (46–80)	62 (44–68)
**Natural Killer cells (CD16^+^CD56^+^)**	67 (62–80) #	61 (53–72)	62 (53–66) #
**CD16^+^CD56^++^**	98 (97–100)	97 (95–99)	98 (93–99)
**CD16^-^CD56^++^**	99 (96–100)	97 (95–99)	98 (97–100)
***Bridging immunity***			
**Natural Killer T cells**	77 (70–89)	70 (61–74)	77 (62–82)
***Adaptive immunity***			
**B cells**			
**Naive (CD10^-^CD27^-^)**	38 (35–46)	36 (32–41)	35 (24–40)
**Memory (CD10^-^CD27^+^)**	45 (39–52)	43 (36–46)	41 (19–47)
**Immature transition (CD10^+^CD27^+^)**	74 (61–81)	72 (58–87)	77 (57–82)
**CD4^+^ T helper cells**			
**CD45RO^-^ CXCR3^-^**	27 (25–32) #	25 (20–38)	25 (13–27) #
**CD45RO^-^ CXCR3^+^**	54 (46–88)	49 (40–58)	50 (43–67)
**CD45RO^+^ CXCR3^-^**	34 (31–39)	32 (28–41)	33 (14–37)
**CD45RO^+^ CXCR3^+^**	43 (36–62)	40 (33–54)	47 (33–60)
**CD8+ cytotoxic T cells**			
**CD45RO^-^ CCR7^-^**	29 (11–34)	31 (24–44)	29 (12–33)
**CD45RO^-^ CCR7^+^**	26 (22–48)	24 (19–44)	25 (20–38)
**CD45RO^+^ CCR7^-^**	31 (13–41)	36 (30–44)	30 (7.6–35)
**CD45RO^+^ CCR7^+^**	58 (52–76)	55 (35–80)	58 (52–77)
**Regulatory T cells (CD25^+^CD127^-^)**	23 (21–27)	20 (15–27)	22 (19–28)

Percentage expression of adiponectin receptor 2 on leukocyte subsets of lean controls compared to obese children pre-lifestyle intervention (pre) and post-lifestyle intervention (post). Data are presented as median (interquartile range).

# p<0.05 for lean controls versus obese-post.

**Table 5:** Leptin receptor expression.

	Lean controls	Obese-pre	Obese-post
***Innate immunity***			
**Monocytes (total)**	96 (95–98)	97 (92–98)	97 (94–98)
**CD14^++^CD16^-^**	99 (99–100)	100 (99–100)	100 (99–100)
**CD14^++^CD16^+^**	100 (99–100)	100 (99–100)	100 (99–100)
**CD14^+^CD16^++^**	70 (61–81)	75 (65–84)	77 (77–81)
**Natural Killer cells (CD16^+^CD56^+^)**	8.0 (5.3–12)	8.8 (3.3–18)	6.1 (4.4–10)
**CD16^+^CD56^++^**	7.2 (5.3–9.6)	5.7 (3.0–14)	6.2 (3.0–9.7)
**CD16^-^CD56^++^**	6.6 (5.9–7.7)	5.3 (3.1–7.1)	6.4 (2.9–7.8)
***Bridging immunity***			
**Natural Killer T cells**	11 (5.8–19)	13 (5.2–32)	9.8 (8.7–18)
***Adaptive immunity***			
**B cells**			
**Naive (CD10^-^CD27^-^)**	51 (44–56)	51 (46–58)	47 (46–55)
**Memory (CD10^-^CD27^+^)**	60 (57–65)	63 (54–65)	57 (50–62)
**Immature transition (CD10^+^CD27^+^)**	46 (39–54)	50 (35–57)	46 (39–51)
**CD4^+^ T helper cells**			
**CD45RO^-^ CXCR3^-^**	31 (28–40)	29 (24–41)	28 (24–36)
**CD45RO^-^ CXCR3^+^**	69 (59–93)	62 (50–73)	71 (64–80)
**CD45RO^+^ CXCR3^-^**	26 (22–29)	25 (21–26)	24 (21–29)
**CD45RO^+^ CXCR3^+^**	42 (29–50)	41 (28–49) $	55 (44–66) $
**CD8+ cytotoxic T cells**			
**CD45RO^-^ CCR7^-^**	22 (16–26)	22 (17–34)	25 (19–29)
**CD45RO^-^ CCR7^+^**	41 (31–52)	39 (31–52)	38 (32–48)
**CD45RO^+^ CCR7^-^**	21 (19–32)	24 (19–32)	28 (24–31)
**CD45RO^+^ CCR7^+^**	65 (56–72)	67 (48–87)	69 (61–80)
**Regulatory T cells (CD25^+^CD127^-^)**	10 (7.8–17)	12 (5.9–15)	13 (11–17)

Percentage expression of leptin receptor on leukocyte subsets of lean controls compared to obese children pre-lifestyle intervention (pre) and post-lifestyle intervention (post). Data are presented as median (interquartile range).

$ p<0.05 for obese-pre compared to obese-post.

Considering the adaptive immune cells, AdipoR1 expression appeared to be low, in general. Only part of the CD4^+^ CXCR3^+^ T-cells and CD8^+^ T-cells and a discrete subset of regulatory T-cells showed AdipoR1 expression. In contrast, AdipoR2 was particularly expressed by B-cells, next to a subset of CD4^+^ CXCR3^+^ T-cells and a subset of CD8^+^ CD45RO^+^ (memory) T-cells. Finally, leptin receptor was predominantly expressed by B-cells, and a subset of CD4^+^ CD45RO^-^ (non-memory) CXCR3^+^ T-cells and of CD8^+^ CD45RO^+^ (memory) CCR7^+^ T-cells and regulatory T-cells (Fig 1, Tables 3, 4 and 5).

Taken together, circulating leukocyte subsets showed distinct adipokine receptor expression profiles. Monocytes generally expressed high levels of all adipokine receptors, while other leukocyte subsets showed a different pattern. NK and iNKT cells predominantly expressed AdipoR2, while B-lymphocytes and CD4^+^ and CD8^+^ T-lymphocyte subsets expressed AdipoR2 as well as leptin receptor. Notably, regulatory T-cells showed little adipokine receptor expression, but a discrete subset of regulatory T-cells expressed a combination of adipokine receptors (Figure in S2 Fig).

### Adipokine receptor expression in lean and obese children

In order to establish differences in adipokine receptor expression between obese children pre and post-intervention and normal weight controls, we studied differences in expression percentages (Tables 3, 4 and 5), as well as median fluorescence intensities (MFI) (S1–S3 Tables). In general, obese children pre and post-intervention and normal weight controls showed similar adipokine receptor expression on leukocyte subsets. In fact, none of the observed differences in adipokine receptor expression between obese children and lean controls remained significant after multiple testing correction.

As an alternative strategy to discriminate between random deviations and potentially relevant differences, we focused on differences in adipokine receptor expression that were consistent in percentages as well as fluorescence intensities. AdipoR1 expression on nonclassical CD14^+^CD16^++^ monocytes was consistently upregulated in percentages and MFI in obese children pre-intervention, compared to lean controls (Table 3 and S1 Table). The other leukocyte subsets did not show consistent differences in AdipoR1, AdipoR2 or leptin receptor expression between obese children and lean controls.

### Circulating adipokines and adipokine receptor expression

**Fig 2:**
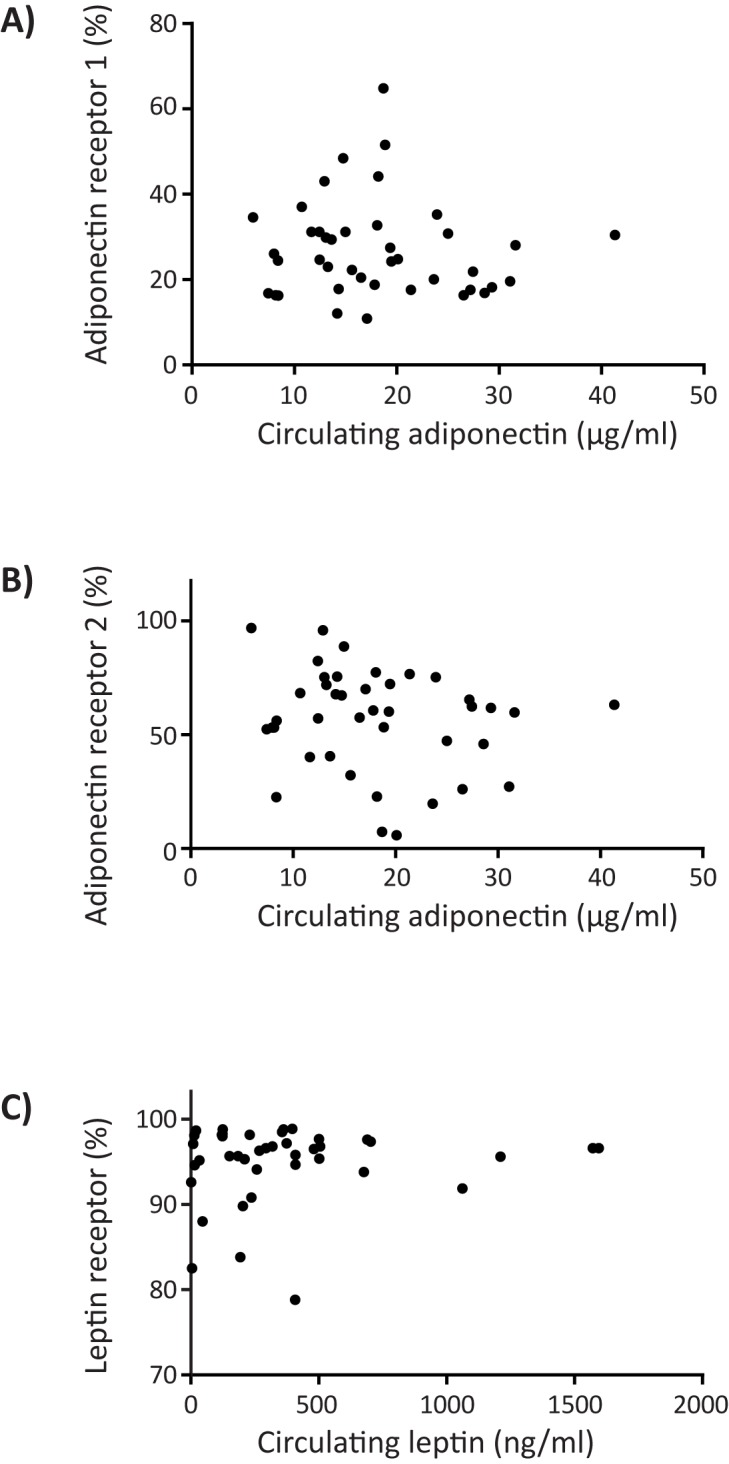
Circulating adipokine levels and monocyte adipokine receptor expression. **(**A) Circulating adiponectin levels versus percentage of AdipoR1 expressing monocytes (Pearson’s correlation coefficient -0.079, p = 0.623). (B) Circulating adiponectin levels versus percentage of AdipoR2 expressing monocytes (Pearson’s correlation coefficient -0.178, p = 0.265). (C) Circulating leptin levels versus percentage of leptin receptor expressing monocytes (Pearson’s correlation coefficient 0.107, p = 0.505).

Finally, we wondered whether high circulating adipokine levels were associated with alterations in adipokine receptor expression, which could modulate adipokine signalling. First, we studied the relationship between circulating adipokine levels and adipokine receptor expression on monocytes. Neither did we observe a correlation between circulating adiponectin levels and AdipoR1/AdipoR2 expression (Fig 2A and 2B), nor a correlation between circulating leptin levels and leptin receptor expression (Fig 2C, Fig B in S3 Fig). Second, screening other leukocyte subsets did not yield a correlation between circulating adipokine levels and adipokine receptor expression either (Fig A in S3 Fig).

## Discussion

Over the last decades, enhanced circulating leptin levels and decreased adiponectin levels emerged as one of the mechanisms underlying the cardiometabolic sequelae in obesity [4,5,11]. This study aimed to establish the expression of adiponectin and leptin receptors on circulating immune cell subsets in obese children and normal weight controls, since adipokine receptor expression on circulating leukocytes can modulate adipokine signalling and obesity-induced systemic inflammation [15,16]. Here, we will discuss our two main findings.

First, circulating leukocyte subsets showed distinct adipokine receptor expression profiles, which may partly explain the differential impact of adipokines on leukocyte subsets. Whereas enhanced AdipoR1/R2 expression on the myeloid cell lineage has for example been associated with anti-inflammatory (M2) macrophage polarization and suppression of foam cell formation [26,27], the decreased AdipoR1 expression on classical CD14^++^CD16^-^ monocytes may be involved in their inflammatory fate and pivotal role in the development of cardiovascular disease [28,29]. Another example is the discrete subpopulation of Tregs expressing a combination of leptin receptor and adiponectin receptors (S2 Fig). Considering the role of peroxisome-proliferator-activated receptor γ (PPARγ) induced adiponectin signalling in adipose tissue differentiation of Tregs [30,31], and the pivotal role of leptin in T-cell differentiation [32–34], it is tempting to speculate that circulating leptin and adiponectin receptor positive Tregs represent recirculating adipose tissue Tregs. Taken together, our data provide an exciting starting point for future studies to the role of adipokine receptors in leukocyte differentiation and function.

Second, we observed largely similar leukocyte subset numbers and adipokine receptor expression profiles in obese children and controls. The high naive CD10^-^CD27^-^ B-cell numbers in obese children pre and post-lifestyle intervention appear to be an exception to that. Interestingly, our findings correspond with recent studies observing high naive B-cell numbers in obese adults [35,36]. The high naïve B-cell numbers may be explained by leptin-induced B-cell hyperstimulation, which can impair B-cell function [35,36]. Indeed, B-cell responses to vaccination can be impaired in obesity [35,37]. With respect to the similar adipokine receptor expression profiles of obese children and lean controls, our findings suggest that in childhood obesity, adipokine signalling in circulating leukocytes is primarily modulated by circulating adipokine levels, instead of adipokine receptor expression.

Our study has a few limitations that have to be taken into account. Storage or freeze-thawing of the peripheral blood mononuclear cells may have neutralized differences in adipokine receptor expression between obese children and controls. Notably, differences in adipokine receptor expression between leukocyte subsets were preserved, which argues against significant storage or freeze-thawing effects. Next, while adipokine receptor expression in liver, skeletal muscle, adipose tissue and other tissues plays in important role in adipokine signalling as well, medical ethical reasons precluded tissue collection. Importantly, our observations in circulating leukocyte subsets do not extend to other tissues. Finally, our study may have been underpowered to identify subtle differences in adipokine receptor expression due to relatively small patient numbers. In obese adults, lymphocyte AdipoR1 and AdipoR2 mRNA expression was reduced compared to anorexic adults [38]. Likewise, reduced monocyte AdipoR1 and AdipoR2 protein expression was observed in obese adults with coronary artery disease, compared to obese adults without cardiovascular disease [39]. However, the results in these studies may have been distorted due to the fact that multiple testing corrections were not applied and nor differentiation was made between functionally distinct leukocyte subsets.

In conclusion, our results cannot exclude subtle differences in adipokine receptor expression, but suggest that adipokine signalling in circulating leukocytes in childhood obesity is primarily modulated by altered adipokine levels.

## Supporting information

S1 Fig Gating strategy of the leukocyte subsets. First, forward scatter (FSC) and sideward scatter (SSC) profiles were used to roughly distinguish monocytes and lymphocytes/NK cells. Second, doublet cells were excluded using FSC-Area (FSC-A) and FSC-Height (FSC-H) gating. Finally, leukocyte subsets were gated on their marker expression.
(TIF)

S2 Fig Combined adiponectin receptor 1 and leptin receptor expression on regulatory T cells (Tregs). Focussing on the CD25^hi^CD127^low^CD4^+^ T-cells (regulatory T-cells), the adiponectin receptor 1-positive subset also expresses the leptin receptor.
(TIF)

S3 Fig Circulating adipokine levels and adipokine receptor expression. *(*A) Circulating adiponectin levels versus percentage of adiponectin receptor 2 expressing iNKT cells (Pearson’s correlation coefficient 0.048, p = 0.764), and circulating leptin levels versus percentage of leptin receptor expressing regulatory T-cells (Pearson’s correlation coefficient -0.130, p = 0.416). (B) Circulating adiponectin levels versus median fluorescence intensity (MFI) of adiponectin receptor 1 and 2 expression (Pearson’s correlation coefficient AdipoR1 0.052, p = 0.746; AdipoR2–0.172, p = 0.284), and circulating leptin levels versus median fluorescence intensity (MFI) of leptin receptor expression (Pearson’s correlation coefficient 0.126, p = 0.431).
(TIF)

S1 Table See separately uploaded file. (DOC)

S2 Table See separately uploaded file. (DOC)

S3 Table See separately uploaded file. (DOC)

S1 Data Database adipokine receptor expression. (SAV)

## Abbreviations

AdipoR1/2: Adiponectin receptor 1/2
BMI-SD: Body mass index standard deviation for age and sex
BP: Blood pressure
CVD: Cardiovascular disease
HDL: High-density lipoprotein
iNKT cells: invariant Natural Killer T-cells
LDL: Low-density lipoprotein
MFI: Median fluorescence intensity
NK cells: Natural Killer cells
PBMC: Peripheral blood mononuclear cells
QUICKI: Quantitative insulin sensitivity index
T2D: Type 2 diabetes
