# Supplementary figures and images for: Differential adipokine receptor expression on circulating leukocyte subsets in lean and obese children
Source: PLoS One. 2017 Oct 26;12(10):e0187068. doi: 10.1371/journal.pone.0187068 (PMC5658151; doi:10.1371/journal.pone.0187068)

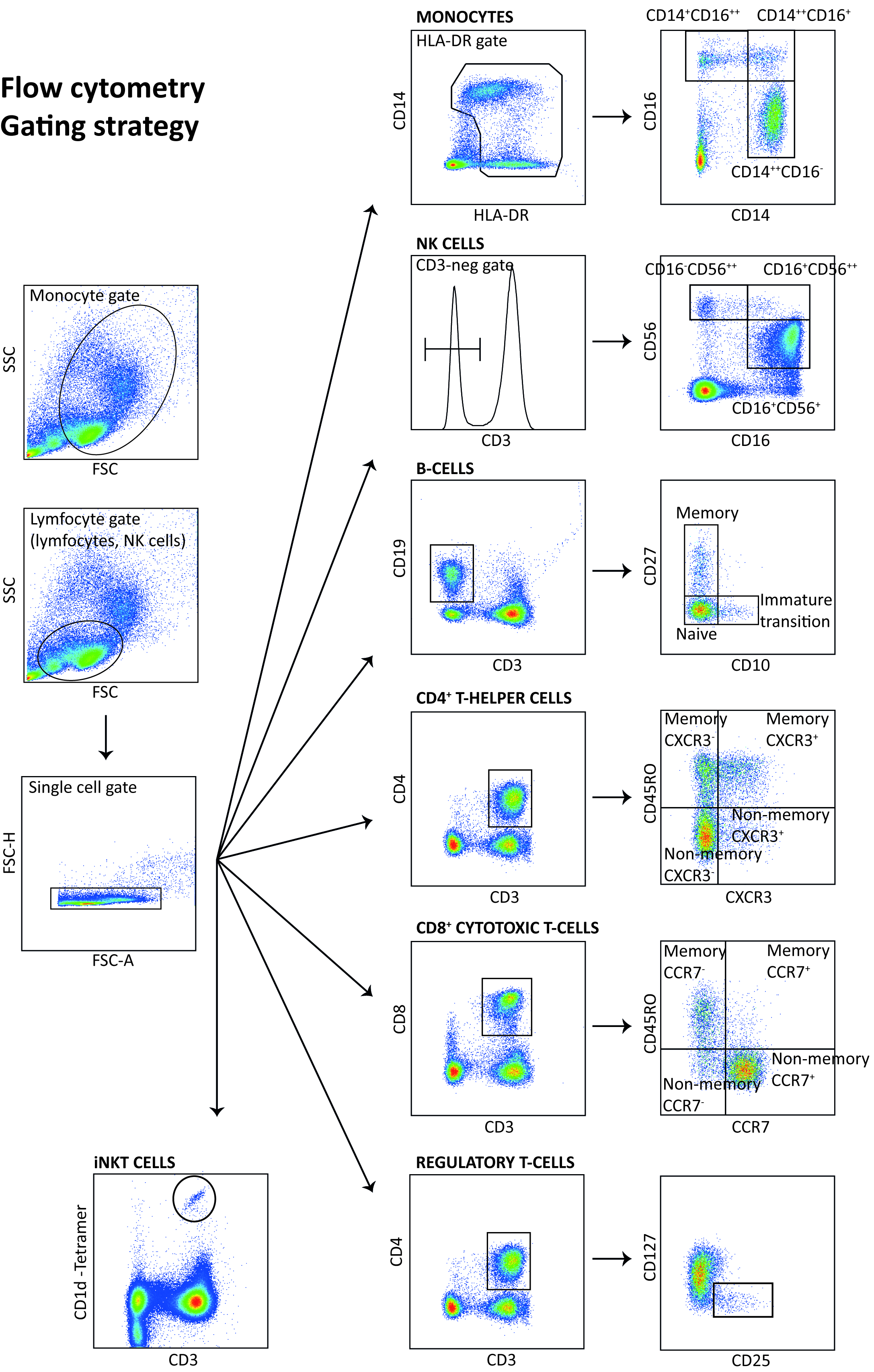

Supplement: S1 Fig — First, forward scatter (FSC) and sideward scatter (SSC) profiles were used to roughly distinguish monocytes and lymphocytes/NK cells. Second, doublet cells were excluded using FSC-Area (FSC-A) and FSC-Height (FSC-H) gating. Finally, leukocyte subsets were gated on their marker expression. (TIF) [file pone.0187068.s001.tif]

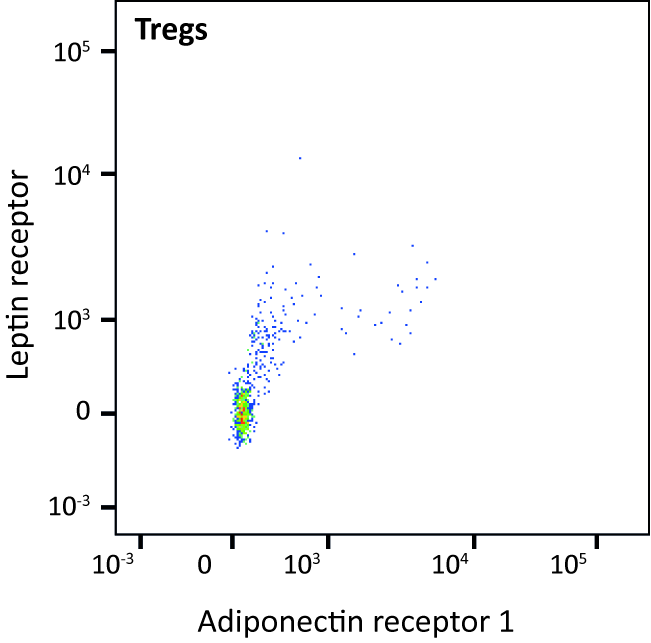

Supplement: S2 Fig — Focussing on the CD25hiCD127lowCD4+ T-cells (regulatory T-cells), the adiponectin receptor 1-positive subset also expresses the leptin receptor. (TIF) [file pone.0187068.s002.tif]

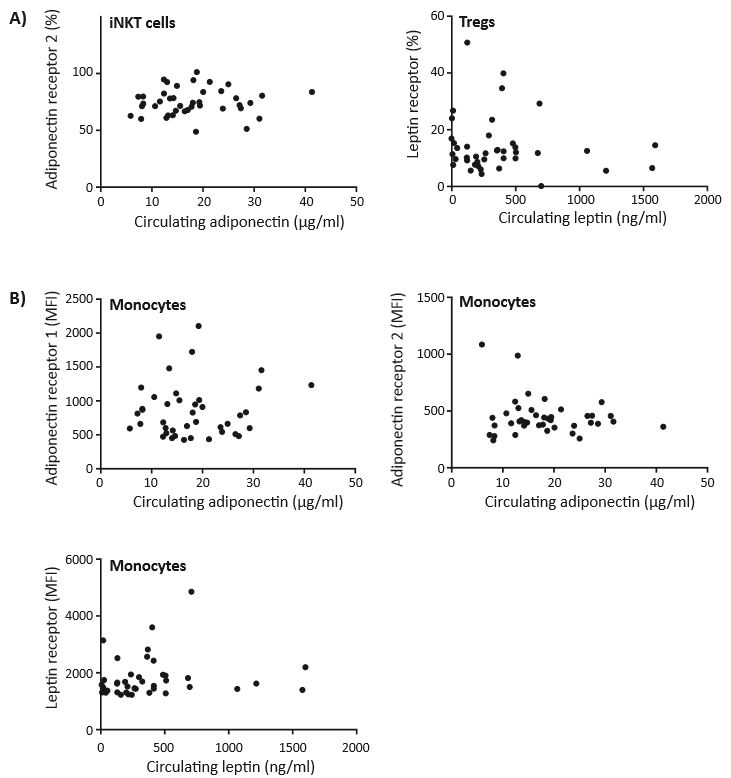

Supplement: S3 Fig — (A) Circulating adiponectin levels versus percentage of adiponectin receptor 2 expressing iNKT cells (Pearson’s correlation coefficient 0.048, p = 0.764), and circulating leptin levels versus percentage of leptin receptor expressing regulatory T-cells (Pearson’s correlation coefficient -0.130, p = 0.416). (B) Circulating adiponectin levels versus median fluorescence intensity (MFI) of adiponectin receptor 1 and 2 expression (Pearson’s correlation coefficient AdipoR1 0.052, p = 0.746; AdipoR2–0.172, p = 0.284), and circulating leptin levels versus median fluorescence intensity (MFI) of leptin receptor expression (Pearson’s correlation coefficient 0.126, p = 0.431). (TIF) [file pone.0187068.s003.tif]
