# Supplementary material for: Differential adipokine receptor expression on circulating leukocyte subsets in lean and obese children
Source: PLoS One. 2017 Oct 26;12(10):e0187068. doi: 10.1371/journal.pone.0187068 (PMC5658151; doi:10.1371/journal.pone.0187068)
Supplement: S1 Table — (DOC) [file pone.0187068.s004.doc]

**S1 Table** Adiponectin receptor 1 expression

|  | **Lean controls** | **Obese-pre** | **Obese-post** |  |
| --- | --- | --- | --- | --- |
| ***Innate immunity*** |  |  |  |  |
| **Monocytes (total)** | 583 (465-769) *# | 931 (582-1306) * | 866 (656-1104) # | |
| **CD14^++^CD16^-^** | 405 (354-522) | 403 (342-479) | 433 (377-461) | |
| **CD14^++^CD16^+^** | 737 (606-1036) | 935 (738-1164) | 835 (745-1063) | |
| **CD14^+^CD16^++^** | 812 (549-1456) * | 1317 (1014-2379) * | 1232 (968-1885) | |
| **Natural Killer cells (CD16^+^CD56^+^)** | 335 (262-453) | 399 (321-510) | 406 (336-517) | |
| **CD16^+^CD56^++^** | 297 (261-396) | 322 (280-366) | 328 (293-390) | |
| **CD16^-^CD56^++^** | 225 (220-270) | 236 (207-266) | 215 (211-265) | |
| ***Bridging immunity*** |  | | | |
| **Natural Killer T cells** | 236 (221-395) | 317 (239-333) | 273 (241-366) | |
| ***Adaptive immunity*** |  | | | |
| **B cells** |  | | | |
| **Naive (CD10^-^CD27^-^)** | 249 (230-279) | 242 (225-264) | 236 (231-264) | |
| **Memory (CD10^-^CD27^+^)** | 228 (219-234) # | 224 (218-232) | 217 (215-225) # | |
| **Immature transition (CD10^+^CD27^+^)** | 224 (197-251) # | 217 (202-240) | 249 (224-496) # | |
| **CD4^+^ T helper cells** |  | | | |
| **CD45RO^-^ CXCR3^-^** | 255 (242-348) | 278 (251-381) | 258 (245-304) | |
| **CD45RO^-^ CXCR3^+^** | 297 (285-383) | 339 (320-457) $ | 308 (299-339) $ | |
| **CD45RO^+^ CXCR3^-^** | 259 (241-332) | 245 (234-370) | 249 (239-314) | |
| **CD45RO^+^ CXCR3^+^** | 279 (263-347) | 299 (262-382) | 286 (262-315) | |
| **CD8+ cytotoxic T cells** |  | | | |
| **CD45RO^-^ CCR7^-^** | 230 (228-233) # | 244 (225-251) | 236 (228-252) # | |
| **CD45RO^-^ CCR7^+^** | 246 (230-306) | 241 (224-356) | 241 (231-231) | |
| **CD45RO^+^ CCR7^-^** | 225 (224-228) | 229 (222-244) | 229 (225-233) | |
| **CD45RO^+^ CCR7^+^** | 279 (273-365) | 354 (255-426) | 310 (275-375) | |
| **Regulatory T cells (CD25^+^CD127^-^)** | 282 (240-314) | 248 (210-277) | 257 (224-271) |  |

Median Fluorescence Intensity (MFI) of adiponectin receptor 1 on leukocyte subsets of lean controls compared to obese children pre-lifestyle intervention (pre) and post-lifestyle intervention (post). Data are presented as median (interquartile range). * p<0.05 for lean controls compared to obese-pre. # p<0.05 for lean controls versus obese-post. $ p<0.05 for obese-pre compared to obese-post.
