# Supplementary material for: Differential adipokine receptor expression on circulating leukocyte subsets in lean and obese children
Source: PLoS One. 2017 Oct 26;12(10):e0187068. doi: 10.1371/journal.pone.0187068 (PMC5658151; doi:10.1371/journal.pone.0187068)
Supplement: S2 Table — (DOC) [file pone.0187068.s005.doc]

**S2** Table Adiponectin receptor 2 expression

|  | **Lean controls** | **Obese-pre** | | **Obese-post** | | |  |
| --- | --- | --- | --- | --- | --- | --- | --- |
| ***Innate immunity*** |  |  | | |  | |  |
| **Monocytes (total)** | 460 (401-518) | 410 (376-535) | 392 (326-434) | | |  | |
| **CD14^++^CD16^-^** | 455 (394-515) # | 405 (369-515) | 378 (321-419) # | | |  | |
| **CD14^++^CD16^+^** | 641 (499-752) | 507 (433-1100) | 491 (414-648 | | |  | |
| **CD14^+^CD16^++^** | 487 (417-553) | 445 (381-561) | 421 (372-482) | | |  | |
| **Natural Killer cells (CD16^+^CD56^+^)** | 370 (358-417) | 351 (296-437) | 352 (291-377) | | |  | |
| **CD16^+^CD56^++^** | 690 (617-858) | 690 (579-753) | 631 (548-726) | | |  | |
| **CD16^-^CD56^++^** | 752 (695-941) | 705 (613-778) | 668 (594-771) | | |  | |
| ***Bridging immunity*** |  | | | | | | |
| **Natural Killer T cells** | 378 (376-602) | 399 (354-418) | 392 (353-419) | | |  | |
| ***Adaptive immunity*** |  | | | | | | |
| **B cells** |  | | | | | | |
| **Naive (CD10^-^CD27^-^)** | 306 (286-318) # | 294 (281-310) | 290 (268-299) # | | |  | |
| **Memory (CD10^-^CD27^+^)** | 308 (289-317) | 301 (282-313) | 305 (271-312) | | |  | |
| **Immature transition (CD10^+^CD27^+^)** | 444 (389-474) | 454 (395-504) | 423 (357-454) | | |  | |
| **CD4^+^ T helper cells** |  | | | | | | |
| **CD45RO^-^ CXCR3^-^** | 294 (282-345) | 297 (273-346) | 283 (278-324) | | |  | |
| **CD45RO^-^ CXCR3^+^** | 348 (328-409) | 345 (314-467) | 341 (308-365) | | |  | |
| **CD45RO^+^ CXCR3^-^** | 308 (293-347) | 304 (280-360) | 298 (282-356) | | |  | |
| **CD45RO^+^ CXCR3^+^** | 333 (305-448) | 332 (292-397) | 331 (291-391) | | |  | |
| **CD8+ cytotoxic T cells** |  | | | | | | |
| **CD45RO^-^ CCR7^-^** | 293 (278-304) | 291 (279 319) | 291 (286-317,) | | | | |
| **CD45RO^-^ CCR7^+^** | 331 (285-901) | 321 (281-530 | 314 (290-698) | | | | |
| **CD45RO^+^ CCR7^-^** | 290 (275-314) | 295 (280 -318) | 291 (286-308) | | | | |
| **CD45RO^+^ CCR7^+^** | 535 (471-1086) | 600 (393-769) | 833 (505-964) | | | | |
| **Regulatory T cells (CD25^+^CD127^-^)** | 297 (286-617) | 297 (263-419) | 320 (280-437) | | |  | |

Median Fluorescence Intensity (MFI) of adiponectin receptor 2 on leukocyte subsets of lean controls compared to obese children pre-lifestyle intervention (pre) and post-lifestyle intervention (post). Data are presented as median (interquartile range). * p<0.05 for lean controls compared to obese-pre. # p<0.05 for lean controls versus obese-post. $ p<0.05 for obese-pre compared to obese-post.
