# Supplementary material for: Differential adipokine receptor expression on circulating leukocyte subsets in lean and obese children
Source: PLoS One. 2017 Oct 26;12(10):e0187068. doi: 10.1371/journal.pone.0187068 (PMC5658151; doi:10.1371/journal.pone.0187068)
Supplement: S3 Table — (DOC) [file pone.0187068.s006.doc]

**S3 Table Leptin receptor expression**

|  | **Lean controls** | **Obese-pre** | **Obese-post** | | |  |
| --- | --- | --- | --- | --- | --- | --- |
| ***Innate immunity*** |  |  | |  | | |
| **Monocytes (total)** | 1451 (1317-1699) | 1670 (1439-2326) | 1705 (1454-1932) | |  | |
| **CD14^++^CD16^-^** | 1467 (1302-1699) | 1677 (1405-2451) | 1532 (1393-1872) | |  | |
| **CD14^++^CD16^+^** | 2236 (1715-2433) | 2524 (1827-4133) | 2227 (2008-2893) | |  | |
| **CD14^+^CD16^++^** | 1188 (1141-1529) | 1402 (1139-1999) | 1467 (1467-1971) | |  | |
| **Natural Killer cells (CD16^+^CD56^+^)** | 387 (370-416) | 384 (362-426) | 388 (367-416) | |  | |
| **CD16^+^CD56^++^** | 380 (350-409) | 365 (342-381) | 374 (356-403) | |  | |
| **CD16^-^CD56^++^** | 376 (356-403) | 377 (365-401) | 378 (350-424) | |  | |
| ***Bridging immunity*** |  | | | | | |
| **Natural Killer T cells** | 483 (403-774) | 577 (462-774) $ | 440 (415-603) $ | |  | |
| ***Adaptive immunity*** |  | | | | | |
| **B cells** |  | | | | | |
| **Naive (CD10^-^CD27^-^)** | 465 (445-487) | 467 (449-479) | 460 (434-480) | |  | |
| **Memory (CD10^-^CD27^+^)** | 521 (502-546) | 527 (492-560) | 507 (481-523) | |  | |
| **Immature transition (CD10^+^CD27^+^)** | 470 (447-500) | 477 (451-529) | 468 (449-516) | |  | |
| **CD4^+^ T helper cells** |  | | | | | |
| **CD45RO^-^ CXCR3^-^** | 417 (409-487) | 410 (405-504) | 419 (407-430) | |  | |
| **CD45RO^-^ CXCR3^+^** | 597 (562-737) | 646 (562-846) | 582 (571-648) | |  | |
| **CD45RO^+^ CXCR3^-^** | 421 (406-441) | 426 (412-529) | 424 (409-451) | |  | |
| **CD45RO^+^ CXCR3^+^** | 504 (444-727) | 527 (437-632) | 577 (502-607) | |  | |
| **CD8+ cytotoxic T cells** |  | | | | | |
| **CD45RO^-^ CCR7^-^** | 410 (398-424) | 402 (388 -464) | 414 (403-445) | |  | |
| **CD45RO^-^ CCR7^+^** | 463 (424-624) | 457 (415-691) | 443 (432-537) | |  | |
| **CD45RO^+^ CCR7^-^** | 412 (395-470) | 429 (400-481) | 436 (417-464) | |  | |
| **CD45RO^+^ CCR7^+^** | 676 (583-753) | 886 (545-1063) | 737 (631-1006) | |  | |
| **Regulatory T cells (CD25^+^CD127^-^)** | 410 (401-494) | 426 (410-489) | 451 (429 -566) | | |  |

Median Fluorescence Intensity (MFI) of leptin receptor on leukocyte subsets of lean controls compared to obese children pre-lifestyle intervention (pre) and post-lifestyle intervention (post). Data are presented as median (interquartile range). * p<0.05 for lean controls compared to obese-pre. # p<0.05 for lean controls versus obese-post. $ p<0.05 for obese-pre compared to obese-post.
